# Exploring Dynamic Brain Functional Networks Using Continuous “State-Related” Functional MRI

**DOI:** 10.1155/2015/824710

**Published:** 2015-08-27

**Authors:** Xun Li, Yu-Feng Zang, Han Zhang

**Affiliations:** ^1^Center for Cognition and Brain Disorders, Hangzhou Normal University, Hangzhou 311121, China; ^2^Zhejiang Key Laboratory for Research in Assessment of Cognitive Impairments, Hangzhou 310015, China

## Abstract

We applied a “temporal decomposition” method, which decomposed a single brain functional network into several “modes”; each of them dominated a short temporal period, on a continuous, “state-” related, “finger-force feedback” functional magnetic resonance imaging experiment. With the hypothesis that attention and internal/external information processing interaction could be manipulated by different (real and sham) feedback conditions, we investigated functional network dynamics of the “default mode,” “executive control,” and sensorimotor networks. They were decomposed into several modes. During real feedback, the occurrence of “default mode-executive control competition-related” mode was higher than that during sham feedback (*P* = 0.0003); the “default mode-visual facilitation-related” mode more frequently appeared during sham than real feedback (*P* = 0.0004). However, the dynamics of the sensorimotor network did not change significantly between two conditions (*P* > 0.05). Our results indicated that the visual-guided motor feedback involves higher cognitive functional networks rather than primary motor network. The dynamics monitoring of inner and outside environment and multisensory integration could be the mechanisms. This study is an extension of our previous region-specific and static-styled study of our brain functional architecture.

## 1. Introduction

Many studies have suggested that our brain is an intricate system [1, 2]. Understanding the spatial and temporal organization of this complex system is of great importance to both basic and clinical neuroscience. Various studies have demonstrated the complexity of the human brain using functional magnetic resonance imaging (fMRI) based on spatiotemporal characteristics with methods such as independent component analysis (ICA) [3] and complex network analysis [4].

Nowadays, a new characteristic of the brain functional networks has been catching researchers' eyes. It is the dynamic functional networks or time-varied functional connectivity which treats the brain functioning as a nonstationary process. Dedicated analysis methods have been proposed. Among various methods, “temporal decomposition” [5–7] does not rely on statistical hypothesis, and it is simple, intuitive, and straightforward. The main idea of this method is that only a small proportion of the time frames corresponding to “suprathreshold signal” of a seed region are utilized and classified into different subgroups or “modes” depending on the spatial similarity of these frames. The modes were interpreted to reflect the intermittent or brief interaction between the seed region and other different brain regions at different time [5–7].

However, in their original paper [7], the analysis was only performed on resting-state fMRI data. The interpretation and the biological meaning of their findings have only been simply discussed because the resting-state brain activity and its functions have been still an elusive concept [8]. In their latter paper [5, 6], they made a methodological alteration in which the fMRI data was totally blindly decomposed without a priori seed region definition. The total data-driven method, in addition to passive resting-state experimental design, has hindered us from further interpretation of the biological meaning and underlying mechanism of this method. To our best knowledge, there has been no study utilizing this method in a task-related experiment. Hence, the resultant dynamic modes could not be compared between different states which are manipulated by researchers. In turn, one could not understand the specific function of the dynamic modes.

In the current study, we applied this method to a continuous, “state-related” task fMRI experiment and sought to discover different dynamic characteristics during different conditions. We hypothesized that, through such an experimental manipulation, the dynamic feature of some modes could be modulated. We specifically conducted a continuous finger force feedback experiment on a group of healthy volunteers. The manipulation was finger force control with true pressure data shown via a visual feedback “real feedback” or with false pressure feedback data “sham feedback.” Previous fMRI studies using the similar experiment have found task activation differences [9] and fractional amplitude of low-frequency fluctuation (fALFF) differences [10] in the DMN between different feedback conditions. Besides, block-designed fMRI studies have reported differences in activation in the contralateral motor cortex during various motor feedback tasks [11, 12]. Neurofeedback can also improve executive functioning in children [13], indicating the alternation of the executive control network (ECN) [14]. Therefore, we chose the PCC, left motor cortex (LMC), and dorsolateral prefrontal cortex (DLPFC) as seed regions, which are key nodes of the DMN, sensorimotor network (SMN) [15], and ECN, respectively.

The first goal was to investigate dynamic characteristics of these brain networks under continuous feedback condition, which could reveal the competition or cooperation among different brain networks during brief time period. The second goal was to investigate the difference in participation of these networks between the real and sham feedback conditions, to further understand the biological mechanisms of the dynamic brain functional organization.

## 2. Materials and Methods

### 2.1. Subjects

There were totally forty-three healthy right-handed adults (22.7 ± 1.6 years, range 19–25; 23 females) participating in this study. Each subject gave written informed consent. The entry criteria included no history of brain injury, neurological illness, and psychiatric disorders. Data from five subjects were excluded due to technical problems or excessive head motion. The remaining 38 subjects (mean age 22.3 ± 1.6 years; 19 females) were further analyzed. All experiments were approved by the Ethics Committee of the National Key Laboratory of Cognitive Neuroscience and Learning, Beijing Normal University, and were conducted in accordance with the Declaration of Helsinki.

### 2.2. Experimental Design

Each participant underwent three 8-minute fMRI scans during resting state, the real feedback state (RF), and the sham feedback state (SF). During resting state, subjects were instructed to keep their eyes closed, remain still, and stay relaxed. The purpose of this session was to allow the subjects to adapt to the fMRI scanning environment. The order of RF and SF sessions was counterbalanced across subjects. In the RF state, the subjects were asked to grip a pressure sensor using the right index finger and thumb. This sensor is one module of an MRI-compatible physiological multichannel analyzer (model MP150, BIOPAC Systems, Inc., Goleta, CA, USA). The sampling frequency was 250 Hz and the pressure sensitivity was 0.01 cmH_2_O. The sensor recorded the pressure in real time via an airtight tube. The pressure was synchronously presented to the participant on a screen. The target force was set at 20 mmH_2_O, which is small enough to reduce the possibility of muscular fatigue. The subjects were asked to continuously maintain the pinch force at the target level. In the SF state, the subjects were also asked to maintain the pinch force at the same level, but with a video of another participant's performance during RF presented. Before the experiment, all the subjects had a short training session. For more details please refer to our previous paper [10].

### 2.3. Image Acquisition

MR images were collected using a Siemens Trio 3-Tesla scanner in the imaging center at Beijing Normal University. The participants lied supine with the head snugly fixed by foam pads to minimize head movement. After localization scanning, three fMRI sessions were conducted using echo-planar imaging sequence with the same parameters: 33 axial slices, repetition time (TR) = 2000 ms, echo time (TE) = 30 ms, flip angle = 90°, thickness/gap = 3.5/0.7 mm, field of view (FOV) = 200 × 200 mm^2^, and matrix = 64 × 64. Then a 3D T1 magnetization-prepared rapid gradient echo (MPRAGE) image was acquired (128 sagittal slices, thickness/gap = 1.33/0 mm, in-plane resolution = 256 × 192, TR/TE = 2530/3.39 ms, inversion time = 1100 ms, flip angle = 7°, and FOV = 256 × 256 mm^2^).

### 2.4. Image Preprocessing

The fMRI data was preprocessed using DPARSFA v2.3 [16] and REST v1.8 [17] based on SPM8 (http://www.fil.ion.ucl.ac.uk/spm/) and Matlab 2013a (the MathWorks, Inc., Natick, MA, USA). Major steps included removal of the first four time points, slice timing correction, head motion correction, spatial normalization to Montreal Neurological Institute (MNI) space, spatial smoothing with a Gaussian kernel (FWHM = 6 mm), temporal filtering (0.01–0.08 Hz), removal of linear and quadratic trends, and regression of covariates, including the global signal, the time series of the white matter and cerebrospinal fluid, and six affine motion parameters [7]. The exclusion criterion for excessive head motion was >2 mm translation or >2° rotation in any direction. We also calculated framewise displacement (FD) as another measure of head motion which could identify possible “bad” frames [18]. These bad frames might affect the following temporal decomposition result. To rule out such an effect, we conducted the following analyses with and without removing the bad frames.

### 2.5. Temporal Decomposition

We chose the PCC, LMC, and DLPFC as regions of interest (ROIs), which are the major nodes of the DMN, SMN, and ECN. Seed regions were 12 mm diameter spheres centered at (0, −53, 26), (−38, −22, 56), and (44, 36, 20) in MNI coordinates, respectively, which were chosen based on previous studies [19–21]. The average time series in each ROI was extracted. A 15% threshold was applied on the time series extracted from the PCC, LMC, and DLPFC; that is, only time frames with BOLD intensity exceeding that threshold were selected for following analysis [7]. This threshold can be set manually, but here we only follow parameter setting of Liu et al. The fMRI frames for all subjects and both RF and SF sessions were chosen and put together and then sorted by *k*-means clustering method [22] based on their spatial similarities. The fMRI frames sorted into the same cluster were averaged and transformed to* Z*-statistical maps by dividing the SE. In paper by Liu et al., after inspection of a series of results with different cluster numbers, the one which showed the best balance between richness and redundancy was reserved. Several automatic cluster number estimation methods were also used but the results were suboptimal [5, 6]. Following their method, we also set this number to be 4, 6, and 8 and then inspected the clustering results separately. We found that the resultant “modes” were clearer when the number of clusters was 4. Therefore, we only showed this result in this paper.

### 2.6. Characterizing Participation of Each Mode and Comparing It between Different States

If a specific mode is dominant, it will show more frequently. We used the occurrence of a specific mode to characterize participation of each mode in the task. We hypothesized that the occurrence of the same modes should be different between real and sham feedback states. We calculated the number of suprathreshold time frames of the four modes for every subject and for RF and SF sessions separately and used the ratio between this number and the total time frames as the occurrence of modes. Paired* t*-tests were performed on the ratios between the real and sham feedback states.

## 3. Results

### 3.1. Dynamic Modes of PCC, LMC, and DLPFC

**Figure 1:**
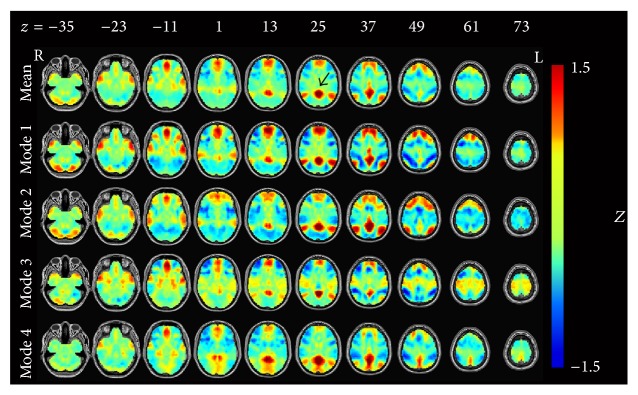
The posterior cingulate cortex- (PCC-) related modes after temporal decomposition. All results were converted to* Z*-maps and arranged by the occurrence frequency. The first line represents the average pattern of the four modes. Black arrow in the first row points out the seed region approximately.

**Figure 2:**
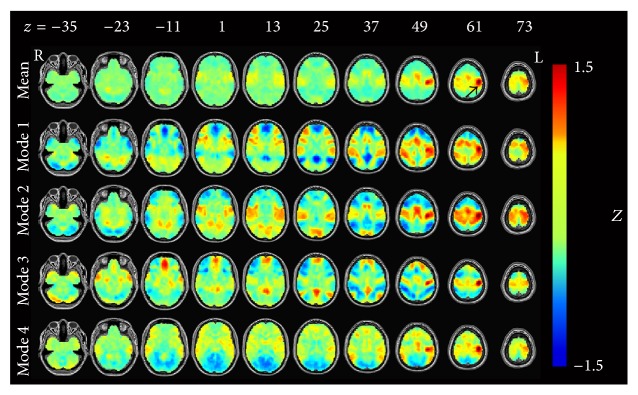
The left motor cortex- (LMC-) related modes after temporal decomposition. All results were converted to* Z*-maps and arranged by occurrence frequency. The first line represents the average result of the four modes. Black arrow in the first row points out the seed region approximately.

**Figure 3:**
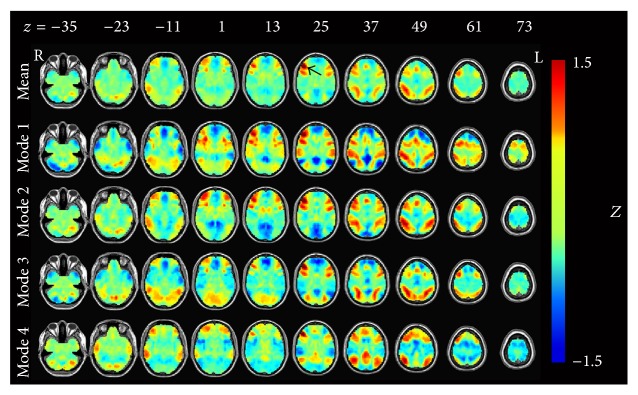
The dorsal lateral prefrontal cortex- (DLPFC-) related modes after temporal decomposition. All results were converted to* Z*-maps and arranged by occurrence frequency. The first line represents the average result of the four modes. Black arrow in the first row points out the seed region approximately.

The temporal decomposition results based on the seed regions of the PCC, LMC, and DLPFC are shown in Figures 1–3. They were ranked by the occurrence of modes despite the task state (i.e., overall occurrence for both RF and SF sessions). The DMN, SMN, and ECN were dominant in these three results. Besides this, several other brain networks also took part in the feedback process. In Figure 1, the average map of the four modes matched well with the DMN, while the spatial pattern of the four modes differed from each other. Mode 1 showed activation of the DMN as well as deactivation of the regions in dorsal attention network (DAN) and ECN [19], which were typical anticorrelated networks demonstrated by Fox and his colleagues [23]. Mode 2 also showed activation of the DMN, but without obvious anticorrelated networks. Besides DMN, the activation of the frontoparietal control network (FCN) [24] was also found in a few upper slices. Mode 3 showed the coactivation of the SMN and DMN, with deactivation of the DAN and ECN. Mode 4 showed the coactivation of the DMN and the visual areas. With all bad frames removed, reanalyses of the temporal decomposition produced similar results (see Figure S1 in Supplementary Material available online at http://dx.doi.org/10.1155/2015/824710). A quantitative differentiation of these modes was further conducted using Dice coefficient to measure the dissimilarity among them, indicating a fundamental difference in spatial pattern between each other (see Supplementary Material and Figure  S2).

In Figure 2, the averaged map shows an obvious pattern of sensorimotor network. However, Mode 1 looked like the combination of the DAN and ECN, with the decreased activity of the DMN. Mode 2 showed the activation mainly in the SMN. Modes 3 and 4 both showed activation of the SMN, but in Mode 3 we also found the pattern of DMN, while in Mode 4 we could have found the decreased activity in visual areas.

The averaged map in Figure 3 shows ECN dominance. Mode 1 shows the anticorrelation between the ECN and the DMN. From Mode 2, we could find the activation of the ECN and FCN and the deactivation of the visual areas. Mode 3 showed the coactivation of the ECN and the visual areas. Mode 4 showed the coactivation of the FCN and DMN.

### 3.2. Different Occurrence Frequency under Different Feedback States

**Figure 4:**
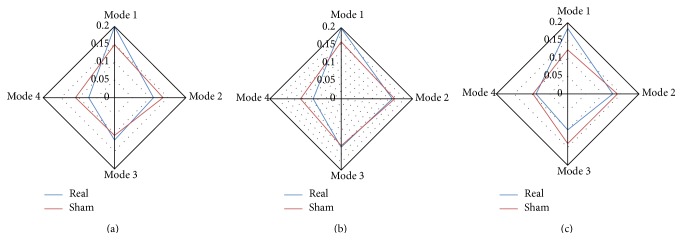
The occurrence frequency of different modes for three seed regions under real and sham conditions. (a) PCC-related modes, (b) LMC-related modes, and (c) DLPFC-related modes.

Figure 4 shows the occurrence frequency of the different modes derived from three different seed regions under real and sham feedback states. The paired* t*-test was performed on occurrence frequency between the two states for every mode. As we performed totally 12 times (3 seed regions × four modes) of paired* t*-tests, we divided a significant *P* value of 0.05 by 12 to perform multiple comparison correction (i.e., use *P* < 0.0042 to achieve the corrected *P* value of 0.05).

**Figure 5:**
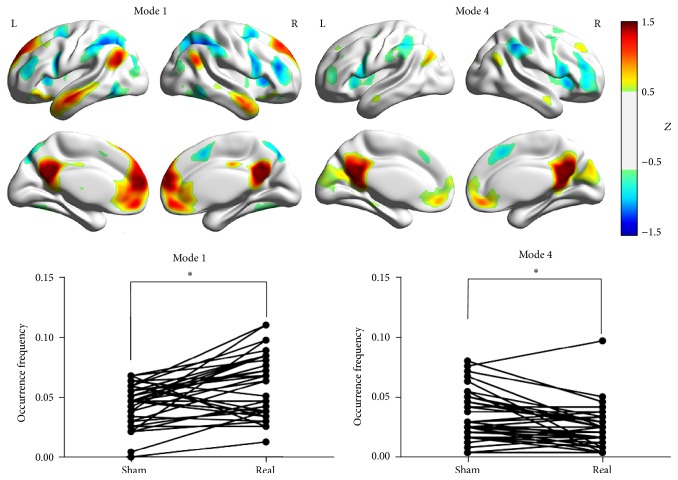
The brain surface view of the PCC-related Mode 1 and Mode 4. For a better view, we set a threshold of *Z* = 0.5 here to separate the brain regions. The paired* t*-test results of these two modes are shown at the bottom.* x*-axis represents two states, while* y*-axis represents the occurrence frequency. The sign of ∗ represents the *P* value lower than 0.05 (corrected).

**Figure 6:**
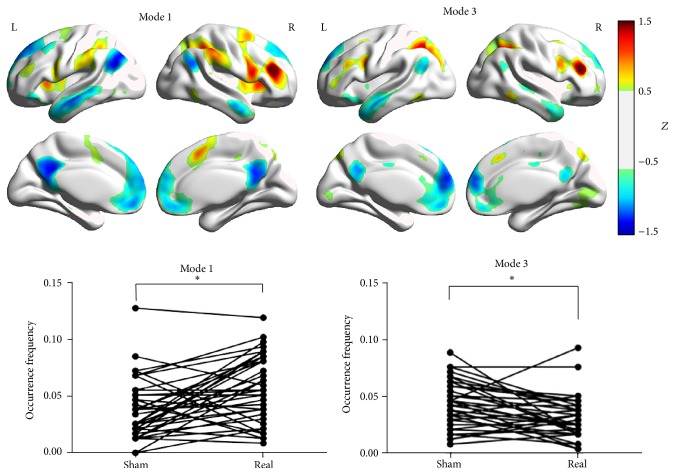
The brain surface view of the DLPFC-related Mode 1 and Mode 3. For a better view, we set a threshold of *Z* = 0.5 here to separate the brain regions. The paired* t*-test results of these two modes are shown at the bottom.* x*-axis represents two states, while* y*-axis represents the occurrence frequency. The sign of ∗ represents the *P* value lower than 0.05 (corrected).

We found that PCC-related Mode 1 (real > sham, *P* = 0.0003) and Mode 4 (sham > real, *P* = 0.0004) were significantly modulated by different feedback states. However, for LMC-related modes, no difference was found. For DLPFC-related modes, Mode 1 (real > sham, *P* = 0.0011) and Mode 3 (sham > real, *P* = 0.0031) were found to have different occurrence between two states. The surface view of these modes as well as their occurrence frequency for all subjects under RF and SF states is plotted in Figures 5 and 6.

## 4. Discussion

In this study, we performed a new method, investigated the dynamic characteristics of brain networks on finger force feedback fMRI data, and had two main findings. Firstly, we found that the DMN, SMN, and ECN did not act independently. Instead, several other networks or brain regions are coactivated or competed with them, which formulated different modes from a brief time period. Secondly, different modes participated differently during RF and SF states. Such a difference was not in spatial pattern but in temporal information (i.e., occurrence frequency).

### 4.1. Biological Meaning of Different Modes

We found that different modes did not represent different brain networks; they were more likely to be combinations of several networks [25]. Previous study has already shown a complex and dynamic functional architecture of the PCC [26]. This may explain the variety of the modes derived from the time frames when PCC was active. From PCC-related Mode 1 and DLPFC-related Mode 1, we found an obvious competition between the DMN and ECN. This might reflect the reciprocal relationship between internal monitoring or self-reference and high-order cognition-related functions. Interestingly, though this competition occupied most of the time, PCC-related Mode 2 and DLPFC-related Mode 4 indicated that these two networks could be coactivated sometimes. PCC-related Mode 2 validated the finding from Spreng et al. [24] in which they found that the FCN acted as regional convergence zones that functionally interact with both default and dorsal attention regions during cognitive tasks [24]. Another interesting finding is that, for all seed regions, there were always several modes having a close relationship with the visual areas. Although such a relationship was not predominant throughout the time course, it occurred at some brief time period as discovered by the dynamic analysis method we used. This might be due to the intensive visual feedback during the tasks. We also compared the temporal decomposition result with that derived from a widely used functional connectivity analysis method, ICA, using MICA toolbox (http://www.nitrc.org/projects/cogicat/, [27]). The two results were quite different (see Supplementary Material and Figure  S3), indicating that conventional stationary analysis method could not find dynamically interacted brain networks.

### 4.2. Different Occurrence of the Modes under Different Feedback States

We found all of the modes based on both RF and SF feedback fMRI data. However, the same modes did not mean the same occurrence. The paired* t*-test results showed significant differences in occurrence frequency of the PCC-related Modes 1 and 4 as well as the DLPFC-related Modes 1 and 3. The PCC-related Mode 1 and the DLPFC-related Mode 1 (they both reflected “default mode-executive control competition”) appeared more frequently during RF, while the other two modes (“default mode-visual facilitation” or “executive control-visual facilitation”) appeared more frequently during SF. The “default mode-executive control competition” might be the basis of finger force feedback, since this type of feedback involves both self-monitoring and executive controlling. The “default mode/executive control-visual facilitation” might be caused by the unmatched self-produced finger force and visual feedback. We should note that although subjects had realized that the finger force curve shown in the screen was not produced by themselves, they might still try to follow the curve in some instant. This speculation was further validated by inquiring the subjects after the experiments. No significant difference in occurrence frequency was found for the SMN-related modes. We speculated that, for motor network, there was no difference in task demand between RF and SF states, since both tasks required similar continuous finger force pressure holding.

### 4.3. Limitations

Our study has several limitations. First, the RF and SF data were put together into temporal decomposition, which has to be based on a hypothesis that the spatial pattern of the modes should be similar during two states. However, we had no direct evidence to support this point. Second, the combination and competition of different brain networks need to be extensively investigated, together with integration of other data, such as behavioral performance and physiological signal recording, to facilitate the interpretation. Third, we only simply averaged the time frames in the same category and this may eliminate the information of the occurrence orders (i.e., precise timing information). Such information may be more informative, which needs further investigation. Fourth, as the intersubject differences or variability is of great importance in neuroscience specially resting-state studies, we also did the temporal decomposition for a randomly selected subject. However, with blurred patterns (result not shown here), the four modes derived from this subject had no correspondence with those derived from all the subjects. We admitted that the application of this method at individual level is currently not applicable. High individual variability should be expected. However, such an individual variability could be an interesting topic for correlation with individual behavior data. Finally, the spatial and temporal resolution will affect the result, especially for dynamic functional connectivity studies like ours. As clustering is based on spatial similarity, the higher spatial resolution is the more accurate spatial clustering result that can be achieved. Besides, the increase of temporal resolution will produce more frames for the following clustering, and one might detect much more transient functional connectivity patterns. In the future, an imaging sequence with both high spatial and temporal resolution should be used, that is, multiband echo-planar imaging [28].

## 5. Conclusions

In this study, we performed temporal decomposition analysis on a group of subjects who performed finger force feedback tasks and revealed different modes combined by various brain networks. The occurrence frequency of several modes showed difference between two feedback states. These findings could help us better understand the dynamics of the functional integration of our brain.

## Supplementary Material

To investigate head motion influence, we removed bad frames with excessive framewise displacement and repeated our analyses. The results were similar. We also made quantitative discrimination among different modes and found that they had little overlap. In addition, we compared the temporal decomposition result with that derived from independent component analysis and found that the two methods had fundamental differences.
