# Supplementary material for: Exploring Dynamic Brain Functional Networks Using Continuous “State-Related” Functional MRI
Source: Biomed Res Int. 2015 Aug 27;2015:824710. doi: 10.1155/2015/824710 (PMC4564637; doi:10.1155/2015/824710)
Supplement: Supplementary file 1 — To investigate head motion influence, we removed bad frames with excessive framewise displacement and repeated our analyses. The results were similar. We also made quantitative discrimination among different modes and found that they had little overlap. In addition, we compared the temporal decomposition result with that derived from independent component analysis and found that the two methods had fundamental differences. [file 824710.f1.docx]

**Exploring dynamic brain functional networks using**

**continuous “state-related” functional MRI**

**Supplementary information**

*Head motion correction by using framewise displacement (FD)*

To evaluate the potential influence of bad frames, we used a method proposed by Power et al. (2012) named framewise displacement (FD). K-means clustering was performed again on frames identified by PCC time series after removing the frames with FD larger than 0.2 (Power et al., 2013). From all subjects’ data, 9.04% frames were identified as bad frames. The final result was shown in Figure S1. The modes were almost the same as Figure 1. The spatial correlation between the results with and without bad frame removal for mean map and modes 1-4 were 0.9960, 0.9987, 0.9989, 0.9988, 0.9982, respectively. Besides, the fractions of these four modes were similar with and without elimination of bad frames. However, to maximally eliminate suspicions, we recommended remove bad frames before temporal decomposition.


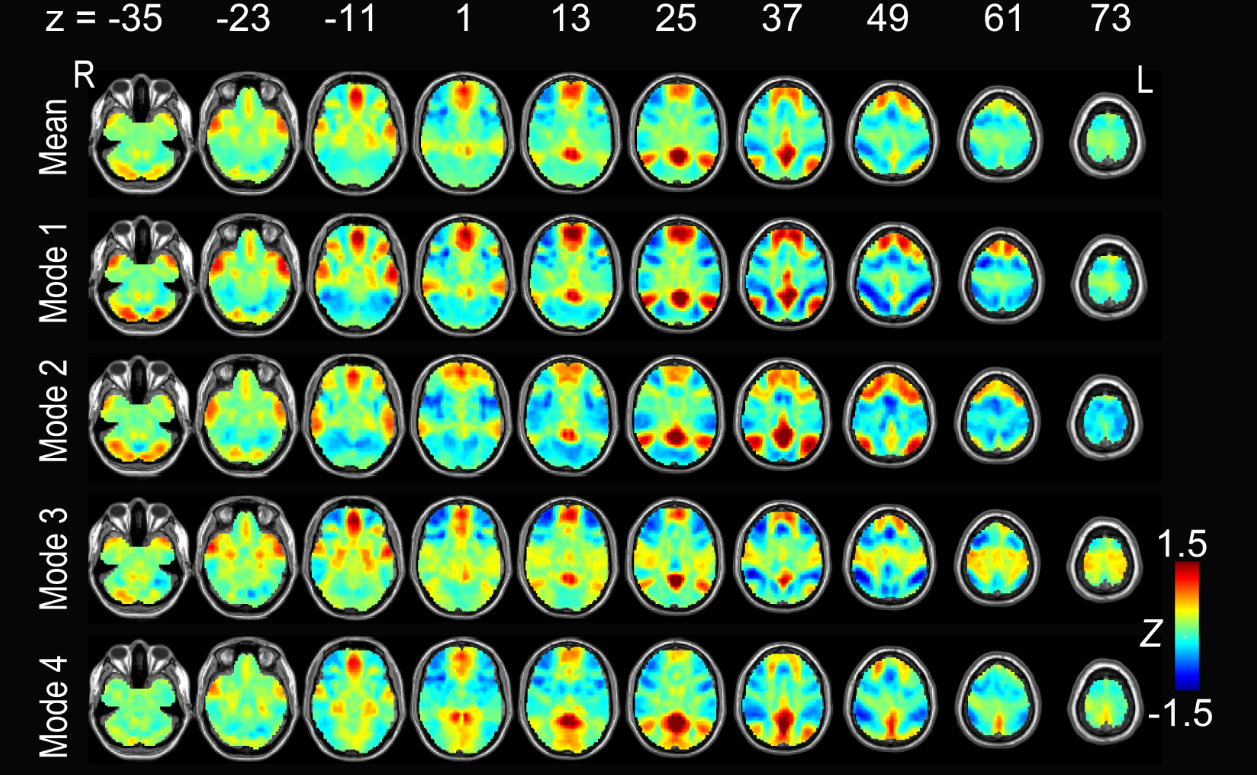


Figure S1. Posterior cingulate cortex (PCC)-related modes after removing bad frames from data. All results were converted to *Z*-maps, and arranged by the occurrence frequency. The first line represents the average pattern of the four modes.

*Quantitative differentiation among different modes*

There could be a concern that the four modes generated by clustering did not differentiate from each other. From visual inspection, the four modes related to the PCC (Figure 1) were quite similar despite of several differences. To quantitatively differentiate among them, we took the PCC-related modes as an example and calculated Dice coefficients which measured the percent overlap between pairs of the four modes. We firstly transformed the four modes into binary maps (z threshold was < -0.5 or > 0.5) and then calculated the Dice coefficients. Figure S2 shows the overlap between the PCC-related Mode 1 and the other 3 modes. The Dice coefficients were 0.4881, 0.4860, and 0.4903, respectively. The Dice coefficients for Mode 2&3, and Mode 2&4 were 0.3171 and 0.3707. The Dice coefficient for Mode 3&4 was 0.4915. This indicated that at least a half of each mode was different from each other, and the overlap among them was only poor-to-moderate.


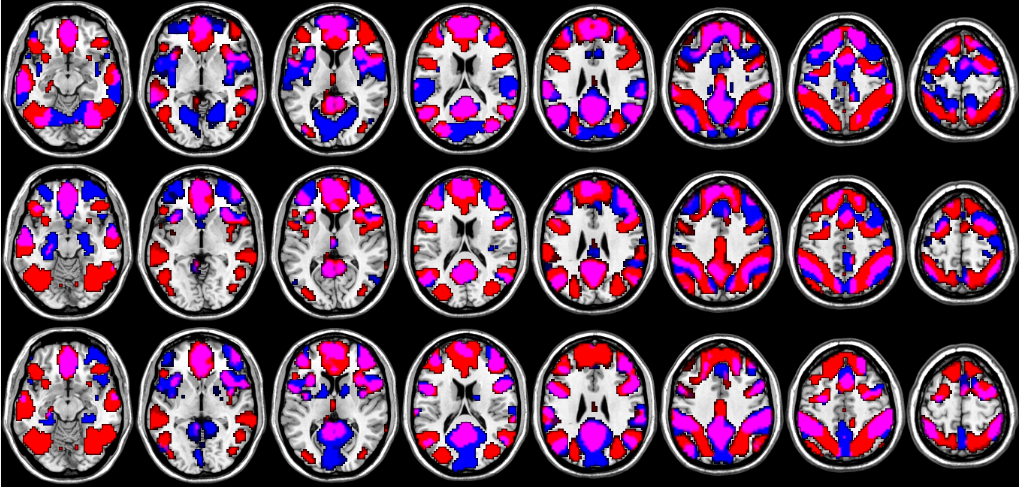


Figure S2. Overlap of the PCC-based Mode 1 with the PCC-related Modes 2, 3, and 4 (the regions shown in the figure have z < -0.5 or z > 0.5).

*Comparison with ICA-derived modes*

To compare the current method with previously widely used network modeling method, MICA toolbox (www.nitrc.org/projects/cogicat/) was adopted to conduct temporally concatenated group ICA on the same data. Total component number was set to be 30. In MICA, group ICA was conducted for 100 times, each with different initial values and data concatenation orders to avoid inconsistency problems (Zhang et al., 2010). As we took PCC’s connectivity pattern as an example, a sphere with center at [0, -53, 26] and radius of 6 mm was generated as ROI. For each components derived from ICA, the average z score within this ROI was calculated. The first four components which had the largest z scores were identified from all 30 components, and were shown in Figure S3. The first three ICA-derived components covered part of the DMN and the last one was dorsal attention network (with the PCC not strongly activated). These results were quite different from those obtained by temporal decompositions based on visual inspection. In ICA result, the PCC only showed coactivity with DMN regions, rather than the sensorimotor areas or visual areas which showed coactivity in temporal decomposition-based result. The reason for such differences is that ICA is a method focusing on stationary functional connectivity based on “spatial independent” hypothesis whereas the temporal decomposition reveals dynamic functional connectivity and it is hypothesis-free.


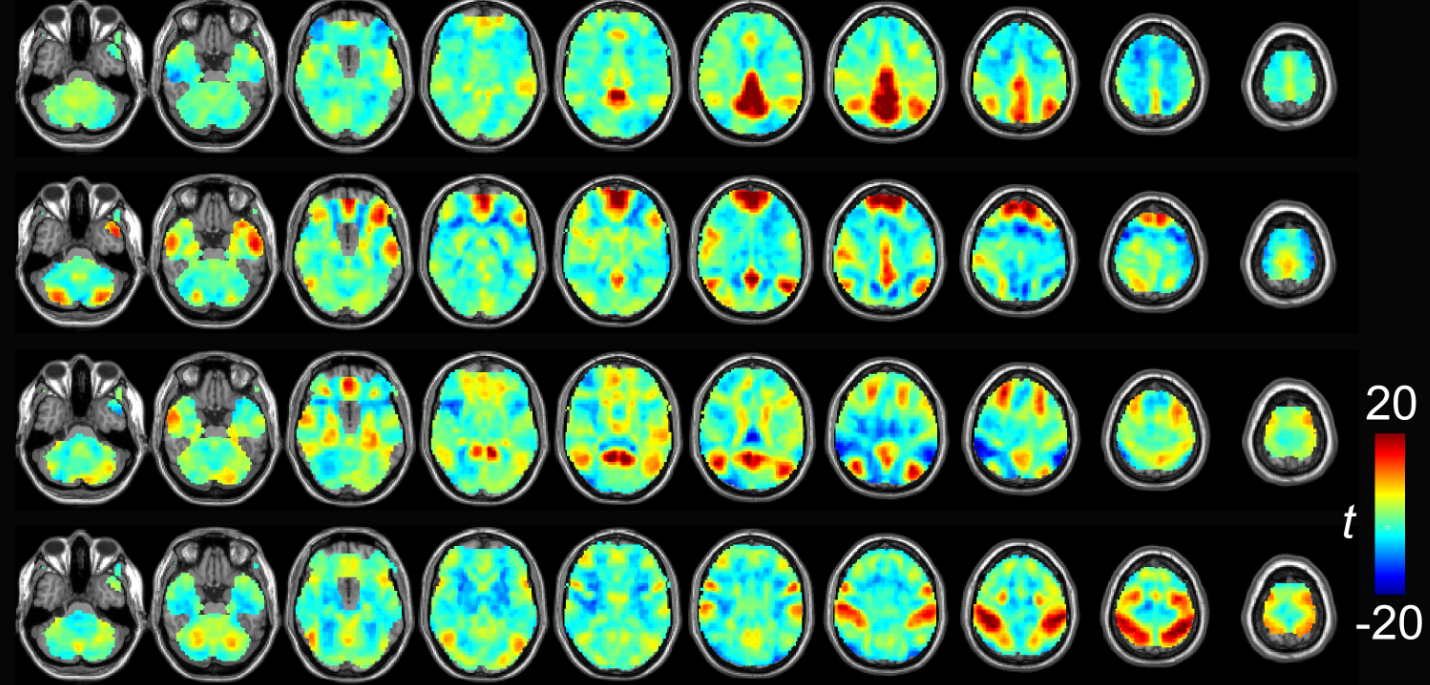


Figure S3. The first four ICA-derived components with the largest z scores in PCC. The results were t maps derived from one-sample t tests. To facilitate comparisons we did not set any threshold on these maps.

*References*

Power, J. D., Barnes, K. A., Snyder, A. Z., Schlaggar, B. L., & Petersen, S. E. (2012). Spurious but systematic correlations in functional connectivity MRI networks arise from subject motion. NeuroImage, 59(3), 2142–2154.

Power, J. D., Barnes, K. A., Snyder, A. Z., Schlaggar, B. L., & Petersen, S. E. (2013). Steps toward optimizing motion artifact removal in functional connectivity MRI; a reply to Carp. NeuroImage, 76, 439–441.

Zhang, H., Zuo, X.-N., Ma, S.-Y., Zang, Y.-F., Milham, M. P., & Zhu, C.-Z. (2010). Subject order- independent group ICA (SOI-GICA) for functional MRI data analysis. NeuroImage, 51(4), 1414–1424.
